# Corneal Nerve Abnormalities in Chronic Central Serous Chorioretinopathy

**DOI:** 10.1016/j.xops.2025.100931

**Published:** 2025-08-29

**Authors:** Jean-Louis Bourges, Bastien Leclercq, Francine Behar-Cohen

**Affiliations:** 1 Department of Ophthalmology, Cochin Hospital, Assistance Publique-Hôpitaux de Paris (APHP), Paris Descartes School of Medicine, Université Paris Cité, Paris, France; 2 Centre de Recherche des Cordeliers, INSERM UMRS 1138 Team 17, Paris, France; 3 Department of Ophthalmology, Foch hospital Suresnne, Suresnes, France

**Keywords:** Cornea, Central serous chorioretinitis, Confocal microscopy, Nerves, Dysautonomia

## Abstract

**Objective:**

Central serous chorioretinopathy (CSCR) is associated with abnormal neural regulation of choroidal blood flow and with systemic dysautonomia. Although choroidal neuropathy has been observed in rodent models of pachychoroid, its presence in human chronic CSCR remains unclear and difficult to examine. Because the long ciliary nerves send fibers to both the cornea and the choroid, this study used in vivo confocal microscopy to examine corneal nerve fiber (CNF) morphology in chronic CSCR patients compared with age-matched controls.

**Design:**

The case-control study included chronic CSCR and control participants without signs or symptoms of corneal disease to detect the presence of nerve abnormalities.

**Subjects:**

The study analyzed corneal nerves in 15 chronic CSCR patients and 11 control subjects with subfoveal thickness ≤360 μm.

**Methods/Testing:**

In vivo confocal microscopy was used to image and systematically analyze the subepithelial area, Bowman layer (BL), anterior stroma, intermediate stroma, and deep stroma. Corneal nerve fiber abnormalities were scored according to their types, location, and number in the different layers by 2 graders in a blind manner. Corneal nerve fibers were quantified in the sub-basal nerve plexi in both groups (chronic CSCR and controls), and subfoveal choroidal thickness was measured using OCT enhanced depth imaging mode.

**Main Outcome Measures:**

Scores of CNF abnormalities in all layers and quantification of CNF in the sub-basal plexi. Statistical difference between CSCR and controls.

**Results:**

In the control group, 8 of 11 individuals showed no CNF abnormalities, whereas 3 had moderate abnormalities in specific corneal layers. In contrast, nearly all CSCR patients exhibited significant CNF abnormalities, including hypertrophic subepithelial nerve plexi, loops, neuromas, cell clusters, dendritic cells activation, and nerve rarefaction. In addition, quantification of BL innervation shows a significant reduction in CSCR patients, indicating corneal nerve abnormalities.

**Conclusions:**

In vivo confocal microscopy reveals morphological nerve abnormalities in chronic CSCR patients, potentially reflecting underlying choroidal neuropathy and subsequent blood flow dysregulation. These findings suggest corneal nerve changes may serve as a noninvasive marker warranting investigation into shared neuropathic mechanisms. Further studies are needed to confirm these preliminary findings and assess their prognostic value.

**Financial Disclosure(s):**

The authors have no proprietary or commercial interest in any materials discussed in this article.

Central serous chorioretinopathy (CSCR) is a prevalent condition among patients exhibiting a pachychoroid phenotype, which is characterized by delayed choroidal filling, the dilation of choroidal veins, and effacement of the choriocapillaris.^1^ This condition is frequently associated with alterations in the overlying retinal pigment epithelium,^1^, ^2^, ^3^ which alterations can compromise the integrity of the barrier, resulting in the accumulation of subretinal fluid. The pathogenesis of pachychoroid is multifactorial, involving genetic predisposition,^4^ venous overload,^5^ scleral rigidity, and neural deregulation of choroidal blood flow.^6^ Indeed, the choroidal blood flow is under the control of the autonomous nervous system^7^^,^^8^ and several studies have brought evidence of systemic dysautonomia in CSCR patients using heart rate variability^9^^,^^10^ and pupillometric responses^11^ to quantify the function of the autonomous nervous system. More recently, a correlation between choroidal thickness and heart rate variability was demonstrated,^12^ supporting the hypothesis that autonomous nervous system dysregulation contributes to the abnormal choroidal vascular phenotype observed in pachychoroid spectrum diseases.

The choroid's functions, including blood flow and thermoregulation, are controlled by a dense neural network comprising sensory input from the trigeminal ganglion, sympathetic input from the superior cervical ganglion, and parasympathetic input from the pterygopalatine ganglia, reaching the eye through the short and the long ciliary nerves, as well as nerve plexus accompanying the ciliary arteries.^7^^,^^13^ We have previously described the innervation of arteries, veins, and capillaries in both human and rodent choroids, finding a calcitonin gene related peptide-positive nerve network that interacts with choroidal cells, including macrophages and mast cells, suggesting a role in neurogenic inflammation.^6^ Three types of fibers (sympathetic, parasympathetic, and sensory) originating from the suprachoroidal ciliary nerves form the limbal plexi around the cornea. These limbal plexi subsequently divide into autonomic fibers, which serve limbal vessels, and sensory nerves that penetrate the cornea after losing their myelin sheets. These fibers collectively form a dense axonal network in the upper anterior stroma (AS) and the subepithelial region, known as the subepithelial nerve plexus, whereas the corneal epithelium receives small nerve bundles termed epithelial leashes. The sub-basal nerve plexus (SNP) is a dense network of nerves that is highly recognizable, with a density that decreases with age but remains consistent across both eyes and sexes.^14^ In addition to sensory nerves, autonomous fibers may innervate the corneal epithelium and vascular endothelium in both rodents and humans.^14^^,^^15^ Although the sensory fibers in the SNP are well described, the autonomic innervation of the cornea, especially beyond the epithelial layers, remains incompletely characterized. However, it is important to consider that the sensory fibers innervating the cornea share common anatomical pathways with the autonomic fibers that innervate the limbus, ciliary body, and iris. Indeed, both sensory and autonomic fibers reach the eye via the ciliary nerves to innervate both the posterior and the anterior segments, sharing common neurobiological substrates. Therefore, the structural analysis of anterior segment nerves, particularly the sensory fibers that can be visualized in the transparent cornea, may reliably reflect the integrity and health of choroidal nerves. Indeed, systemic autonomic diabetic neuropathy was shown to be associated with corneal nerve abnormalities.^16^ Figure 1 shows the anatomical organization of ciliary and corneal nerves.

**Figure 1:**
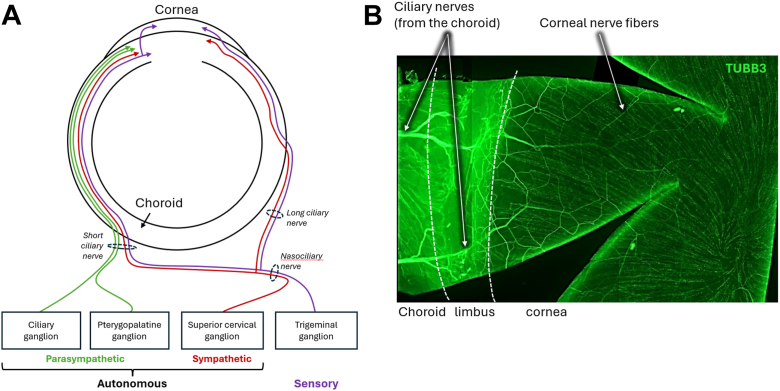
Innervation of the eye's posterior and anterior segments. **A**, A schematic 353 representation of the intraocular innervation. Parasympathetic fibers (from the ciliary and pterygopalatine ganglion), sympathetic fibers (from the superior cervical ganglion), and sensory fibers (from the trigeminal ganglion) form the short and long ciliary nerves. These nerves enter the eye through the sclera and travel toward the anterior segment, innervating both posterior and anterior structures, including the choroid, the iris, the ciliary body, and the cornea. **B**, Photo of a TUBB3 protein (nerve marker) staining in immunohistochemistry, performed on a flat-mounted rat limbal/corneal tissue. Large ciliary nerves coming from the choroid enter the limbus and then form the corneal innervation, highlighting the link between the choroidal and the corneal innervation. TUBB3 = tubbulin 3.

The ocular nervous system's importance has increasingly been recognized in the pathogenesis of ocular surface diseases, such as meibomian gland dysfunction,^17^ limbal deficiency,^18^ and dry eye,^19^ in which in vivo confocal microscopy (IVCM) has been commonly used to reveal signs of nerve damage.^17^ Although no imaging method currently allows clinical visualization of choroidal nerves in vivo, involvement of the choroidal nervous system has been demonstrated in models of age-related macular degeneration^8^ and in a rat model of pachychoroid induced by overexpression of the human mineralocorticoid receptor, where we observed structural signs of choroidal neuropathy.^6^ However, whether choroidal nerves exhibit signs of neuropathy in chronic CSCR patients remains unknown. Because the choroidal nervous system cannot be imaged in the clinic with current techniques and there is a continuum between nerves in the choroid and corneal nerves, we analyzed corneal nerves in patients with chronic CSCR using IVCM and compared them with unaffected controls. Because patients with CSCR showed systemic signs of dysautonomia and choroidal and corneal innervation share the same route, we hypothesize that corneal nerves may reflect choroidal nerve pathology in patients with CSCR.

## Methods

### Design of the Study

Between May 2023 and February 2024, we prospectively enrolled in a case-control study patients with bilateral chronic long-standing CSCR and control volunteers with subfoveal choroidal thickness (SFCT) <360 μm and no signs of pachychoroid to observe their corneal network and detect possible abnormalities. None of the patients or controls exhibited signs or symptoms of any ocular surface diseases. Subjects provided informed consent, and the national institutional review board approved the protocol under Unique Protocol ID# 2022-A02713-40 and ClinicalTrials.gov registration #NCT06466018. Our study adhered to the Declaration of Helsinki.

### Population and Criteria

We analyzed both eyes of each patient, pooling observations under the same patient ID. We included patients over 18 years of age who met the inclusion criteria and matched them with controls for age. The CSCR group consisted of patients with chronic CSCR complicated by epitheliopathy, pachychoroid features, SFCT >390 μm, a history of subretinal fluid, and areas of retinal pigment epithelium alteration >3 disc diameters or multifocal.^20^ The control (CTRL) group comprised volunteers recruited from the same clinical setting, all presenting a SFCT ≤360 μm and carefully screened to exclude any retinal or corneal disease based on medical history, clinical records, and a comprehensive ophthalmic examination performed by an experienced ophthalmologist.

All participants in both groups demonstrated a normal ocular surface for both eyes, as assessed by slit lamp examination and fluorescein testing, with an Oxford grade of 0/0/0 and a tear break-up time >10 seconds. Participants with a history of diabetes, neurotrophic keratitis, or systemic neuropathy were excluded.

### Data Collection

We noticed demographic data such as age and sex. Macula B-scans using OCT enhanced depth imaging mode (Spectralis; Heidelberg) allowed for measurement of SFCT before proceeding with IVCM (HRT3; Heidelberg) corneal exploration. The multilayer module of IVCM acquisition was used. Corneal sensitivity was systematically tested using a fine cotton wisp derived from the tip of a gauze pad, gently applied to the corneal surface, before anesthetizing nociception with chlorhydrate oxybuprocaine eye drops, applied twice at 10-minute intervals. In vivo confocal microscopy was docked toward the central cornea, and acquisition was initiated at the most superficial epithelial layer, proceeding longitudinally toward the corneal periphery to acquire both snapshot frames and multilayer sequences, encompassing the largest possible area. Exploration began centrally, followed by mid-peripheral and paralimbal areas, with a standardized depth and area exploration yielding 240 pictures per patient. We systematically analyzed the corneal nerve network across 5 corneal layers: Bowman layer (BL), subepithelial area (SE), AS, mid-stroma (MS), and deep stroma. We systematically initiated scans from the corneal surface downward. Based on corneal neural network anatomy,^21^ we defined BL as the acellular amorphous layer immediately beneath the basal epithelial cells, the subepithelial layer as the superficial stromal layer directly below BL, the AS as the region within 100 to 150 μm beneath SE, the MS between 150 and 350 μm deep, and the deep stroma as the remaining stromal depth extending down to the endothelium. At least 1 sequential acquisition was performed in the central subepithelial layers, with more than 500 analyzable pictures per patient obtained over the entire corneal area. For each patient, we selected the 4 most representative images demonstrating potential nerve abnormalities from all scanned images for each corneal layer studied; in the absence of evident abnormalities, we selected the 4 images that most accurately represented the normal corneal nerve morphology within the corresponding layer.

Under masked conditions for group and patient characteristics, we semiquantitatively analyzed abnormalities in both groups across the different corneal layers explored by IVCM. Various types of corneal nerve fiber (CNF) abnormalities were identified, following their typical characteristics described previously by other authors, as shown in Figure 1: CNF rarefaction or loops in the BL, neuromas located between the SE and AS layers, scalloped or moniliform CNF within the AS, perineural cell clusters, double-edged CNF between the AS and MS, and activated stromal dendritic cells.^16^^,^^17^^,^^22^^,^^23^

Two senior graders, 1 specializing in corneology (J.-L.B.) and the other in retina (F.B.-C.), independently scored nerve abnormalities per layer as absent (0, no abnormality seen), rare (1, abnormalities observed in less than 10% of all images taken within 1 layer), frequent (2, presence of abnormalities in less than half of the images within 1 layer), or omnipresent (3, presence of abnormalities in half or more of the images within 1 layer), comparing morphological corneal nerves to a gold standard (representative pattern of normal CNF shown in Fig 1).^17^

Concordance of scores between observers was tested, followed by a layer-by-layer analysis of the scores to compare CSCR and CTRL groups. The CNF network of each patient was graded using the system described in Table 1. We arbitrarily assumed that the corneal nerve network was normal if no affected layers or only rare nerve abnormalities were observed in 1 or 2 layers, as agreed by both readers. Finally, we assessed the overall corneal nerve network density.

**Table 1:** Grading System to Qualify the Corneal Nerve Network across the 5 Corneal Layers Analyzed

Score Attributed to a Single Layer	# Layers Concerned	Interpretation Proposed
**1**	n ≤ 2	Normal corneal nerves
2	n = 1	Moderate corneal nerve abnormality
2	n ≥ 2	Severe corneal nerve abnormality
3	n ≠ 0	Severe corneal nerve abnormality

**Grading of corneal nerve abnormalities**

**Normal:** Corneal nerves are considered normal when no abnormalities are observed in any image across all layers, or when abnormalities are present in <10% of images within a single layer and are limited to 1 or 22 layers.

**Moderate abnormalities:** Corneal nerve abnormalities are considered moderate when abnormalities are observed in >10% but <50% of images within a single layer, and only one layer is affected.

**Severe abnormalities:** Corneal nerve abnormalities are considered severe if abnormalities are present in >10% but <50% of images in multiple layers or abnormalities are detected in more than 50% of images in one or more layers.

### Statistical Analysis

Data were tabulated in Excel (Microsoft Office, standard version, 2016) and analyzed using Jamovi 2023 and ad hoc modules (version 2.3; the Jamovi project, https://www.jamovi.org). Given the observational pilot nature of this study, no formal sample size calculation or multiplicity methods were applied. Descriptive statistics were computed for all items. Baseline demographic and clinical characteristics were compared between groups using the Student *t*-test for age, Fisher exact test for sex ratio, and the Mann–Whitney *U* test for SFCT, which measurements did not meet assumptions for the homogeneity of variance. Concordance correlation coefficient values were calculated to test score agreement between the 2 operators (J.-L.B. and F.B.-C.), with <0.90 considered poor, 0.90 to 0.95 moderate, 0.95 to 0.99 substantial, and >0.99 almost perfect. Observations from both eyes were pooled for each patient. Normality of data distribution was assessed using the Shapiro–Wilk test, demonstrating normal distribution in both CSCR (W = 0.972, *P* = 0.891) and CTRL groups (W = 0.990, *P* = 0.997). Homogeneity of variances was confirmed by the Levene test (statistic = 0.004, *P* = 0.951). Although the assumptions required for parametric testing were met, and given the exploratory nature of this study, the Kruskal–Wallis test was used for group comparisons of corneal nerve abnormality, followed by Holm correction to control for multiple comparisons, providing a balanced approach between statistical rigor and preservation of power. Statistical significance was defined as *P* < 0.05, with a confidence level of 95%.

### Quantification of Corneal Nerves within the Sub-Basal Nerve Plexus

Nerve quantification was assessed within the SNP, where the nerves are organized horizontally and distributed uniformly in most images. Four images were quantified for each patient (11 controls and 15 CSCRs) using the semiautomatic tracker of the Simple Neurite Tracer toolbox in the Fiji app.^24^ All fibers were tracked in each image using the A∗ search algorithm included in Simple Neurite Tracer, which automatically detects nerve fiber trajectories. Each fiber was measured, and innervation quantification was determined by summing the individual fiber lengths to obtain a global innervation length per image. To normalize the quantification, only fibers traceable by the algorithm were included. Finally, the total innervation lengths of the 4 images were averaged to obtain a representative value for each patient, which was then compared between CTRL and CSCR patients. Statistical analysis was performed using GraphPad Prism software, beginning with normality and lognormality tests, followed by a Welch *t*-test.

## Results

A total of 15 CSCR patients and 15 CTRL subjects were screened, but IVCM pictures could only be collected from 15 CSCR and 11 CTRL subjects because 2 participants did not proceed with the examination, and 2 had lid spastic reflexes that prevented complete IVCM examination.

Thus, 30 participants were screened: 4 were excluded (2 did not proceed with the IVCM examination, and 2 had lid spasms preventing proper imaging). Twenty-six participants were included in the final analysis: 15 in the CSCR group and 11 in the CTRL group.

The demographic data are provided in Table 2.

**Table 2:** Baseline Demographic and Clinical Characteristics with Statistical Comparisons

Characteristic	CSCR (n = 15)	Control (n = 11)	*P* Value	Statistical Method
Mean age (yrs)	57.2 ± 12.3	49.5 ± 11.5	0.12	Student *t*-test
Sex (M/F)	11/4	6/5	0.419	Fisher exact test
Mean SFCT (μm)	497 ± 111	262 ± 80	<0.001	Mann–Whitney *U* test

CSCR = central serous chorioretinopathy; F = female; M = male; SFCT = subfoveal choroidal thickness.

All corneas were avascular, free from detectable opacity on slit lamp examination, and fully sensitive, with an immediate blink reflex under gentle stimulation present in all cases. The mean subfoveal thickness was 497 μm ± 111 μm [451–543 μm] in the CSCR group and 262 μm ± 80 μm [226–297 μm] in the CTRL group (*P* < 0.0001; Mann–Whitney *U* test, Table 2). The correlation of CNF abnormality scores between readers ranged from good to excellent across all corneal layers (Table 3).^25^ A mean of 658 IVCM pictures was analyzed for CSCR, and a mean of 1171 pictures was analyzed for CTRL.

**Table 3:** Estimated CCC for Nerve Abnormality Scores across Corneal Layers between the 2 Readers

Corneal Layer	CCC	95% CI	
		Lower	Upper
BL	0.93	0.86	0.97
SE	0.85	0.71	0.93
AS	0.8	0.61	0.91
MS	0.77	0.57	0.88
DS	0.75	0.54	0.87

AS = anterior stroma; BL = Bowman layer; CCC = concordance correlation coefficient; CI = confidence interval; DS = deep stroma; MS = mid-stroma; SE = subepithelial area.

**Figure 2:**
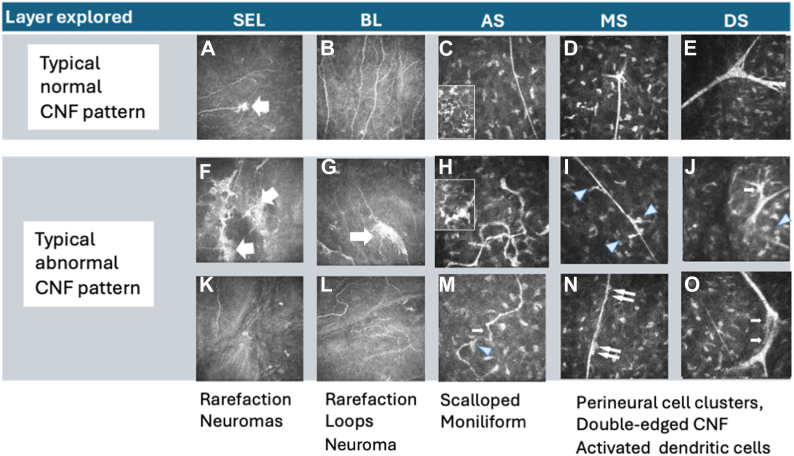
In vivo confocal microscopy images of CNF showing examples of normal patterns and of typical abnormal patterns. Corneal nerve fibers are imaged in 5 corneal layers: SEL, BL, AS, MS, and DS. The arrow in image (**A**) indicates a sub-basal nerve plexus, which is a network of nerve fibers at the interface between BL and the corneal epithelium. Image (**B**) shows typical nerves in the sub bowman layer, (**D**) typical nerves in the mid stroma, and (**E**) typical nerves in the deep stromal layer. Typical abnormalities include rarefaction of the nerve fibers in the SEL and the BL (**G, K,** and **L**). Neuromas are large and irregular in the SEL and BL (**F, G**, and arrows). The scalloped, thickened, and moniliform fibers (**M**; arrow) contrast with the normal CNF, which are usually thin and smoothly curved within the anterior stroma neural network (**C**). Loops correspond to unusual coiling or tortuosity of the fibers (**H**). Activated dendritic cells (**H**; arrowheads) are enlarged, with thick projections located within the stromal matrix (**H**, inset) as opposed to dendritic cells with thin and extended dendritic expansions in the normal pattern (**C**, inset). In the MS and DS, perineural cell clusters (**I** and **J**, blue arrowheads) and double-edged CNF are observed (**N** and **O** arrows). AS = anterior stroma; BL = Bowman layer; CNF = corneal nerve fiber; DS = deep stroma; IVCM = in vivo confocal microscopy; MS = mid-stroma; SEL = subepithelial layer.

In the CTRL group, 8 of 11 individuals exhibited entirely normal CNF according to the gold standard^17^^,^^22^^,^^26^ and as illustrated in Figure 2. Moderate abnormalities were detected in 3 of 11 IVCM images, located anteriorly in the SE (n = 3), BL (n = 2), AS (n = 3), and MS (n = 1). The involvement of no more than 3 layers was observed, according to the neural network grading (Table 1). Three corneas were classified as moderately abnormal; however, no corneas were deemed severely altered.

**Figure 3:**
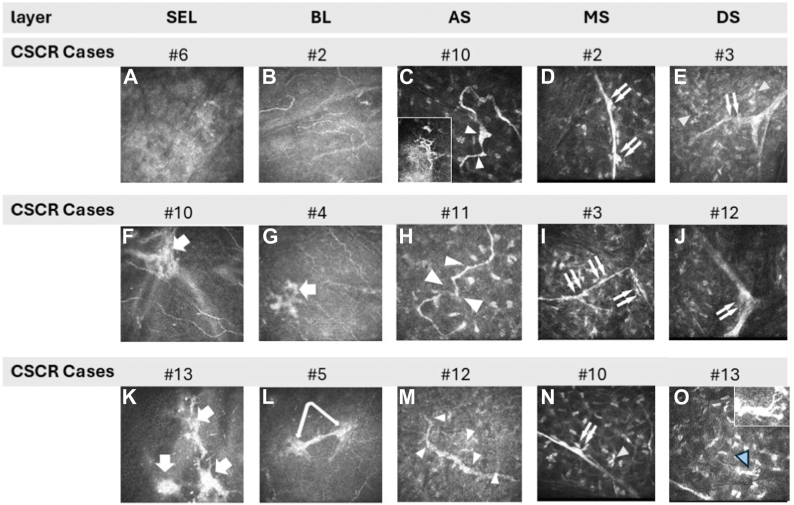
In vivo confocal microscopy frames from CSCR. Examples of images obtained from BL, SEL, and corneal stroma at different depths (AS, MS, and DS). Rarefaction of CNF is observed in BL and SEL (**A, B, F, G, K,** and **L**). The subepithelial nerve plexi (**F, G,** and arrows) appear both enlarged and granulated or elongated (**F** and **G**). Neuromas are observed in SEL and BL (**K, L**, and arrows). Loops and moniliform fibers are observed in the AS (**C, H, M**, and arrowheads). Crossing the MS, the long nerves display swollen and moniliform edges (**D, I,** and **N**). The deep nerve structures are swollen and blistered with isoreflective lumps (double arrows). At the intersection of the large and deep corneal nerve trunks (**E, J,** and **O**), occasional irregular hyper-reflectivity is observed (**N, O**; double arrows). Activate d dendritic cells are detected (blue arrowheads, C and O inset). AS = anterior stroma; BL = Bowman layer; CNF = corneal nerve fiber; CSCR = central serous chorioretinopathy; DS = deep stroma; MS = mid-stroma; SEL = subepithelial layer.

**Figure 4:**
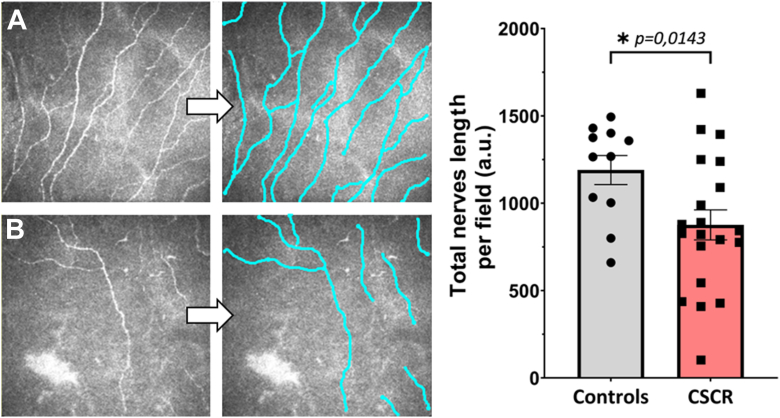
Significant reduction of fibers in the sub-basal nerve plexus in CSCR patients. In vivo confocal microscopy images of the sub-basal nerve plexus were semiautomatically quantified in controls **(A)** and CSCR patients **(B)**. Quantification reveals a significant reduction of nerve length in CSCR patients compared with the control group, as shown in the images and related graph. This reduction represents a sign of neuropathy, consistent with other nerve abnormalities described in this study. CSCR = central serous chorioretinopathy.

In the chronic CSCR group, a single individual was considered normal, with mild CNF alterations. The remaining patients exhibited moderate (n = 2) to severe (n = 12) nerve disorders, with a highly significant difference compared with the CTRL group (*P* ≤ 0.001). The superficial and mid layers exhibited a higher prevalence of alterations compared with the deep layers or the CTRL (*P* ≤ 0.005), with at least ≥2 layers displaying subnormal patterns. In the BL, the presence of twisted and rarefied nerve fibers was observed, with these fibers forming loops of unusual size on occasion. Furthermore, the subepithelial nerve plexus exhibited multiple areas of hypertrophic patterns, accompanied by waved nerve edges that are suggestive of potential neuroma features in both AS and MS nerves (Figs 2 and 3). To quantify the abnormal corneal innervation, semiautomatic tracking and measuring of nerve fibers were assessed in the SNP close to the BL (Fig 4). Chronic CSCR patients display a significant reduction of nerve length compared with the control, which constitutes a highly suggestive sign of corneal nerve abnormalities, consistent with other corneal nerve abnormalities described in this study (Fig 4).

**Figure 5:**
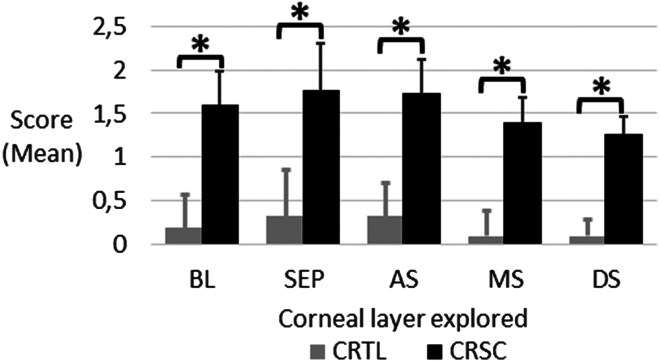
Mean scores of corneal nerve abnormalities observed by IVCM from CTRL and CSCR. ∗: significant difference, Kruskal–Wallis test; Holm corrected *P* value < 0.001. AS = anterior stroma; BL = Bowman layer; CSCR = central serous chorioretinopathy; CTRL = control; DS = deep stroma; IVCM = in vivo confocal microscopy; MS = mid-stroma; SEP = subepithelial nerve plexus.

A single CSCR subject was deemed to be within normal limits with only mild alterations to nerve fibers. The remaining subjects exhibited a range of disorders, from moderate in 2 cases to severe in 12 cases, as illustrated in Figure 3. A statistically significant difference was observed in the extent of nerve alterations across all layers examined, with CSCR demonstrating more significant changes compared with CTRL (*P* ≤ 0.001, Fig 5). Although nerve fibers appeared dense in the CTRL group, almost half of the CSCR patients (n = 7/15) demonstrated marked and diffuse rarefaction. A comparison of the superficial and mid corneal layers with the deep layers revealed a higher prevalence of alterations in the former (Mann–Whitney; *P* ≤ 0.005). It is noteworthy that endothelial cell patterns remained normal in both groups.

With regard to the types of nerve abnormalities observed, it was found that reduced nerve density and moniliform nerve fibers were specific to the pathological condition, with prevalence rates of 53% and 100%, respectively. These abnormalities were not observed in the CTRL groups. Neuroma and scalloped nerve fibers were observed in 9% of the control corneas and in 93% and 92% of the chronic CSCR patients' corneas, respectively, suggesting they are also highly specific to the pathological condition. After applying Holm correction for multiple comparisons, significant differences remained for low nerve density, neuroma, scalloped fibers, moniliform/bulb-like nerve structures, and dendritic cell activation, whereas differences in loops, cell clusters, and double-edged fibers were no longer statistically significant. With the exception of double-edged fibers and perineural clusters, all other abnormalities exhibited a significantly higher frequency in chronic CSCR cases compared with the CTRL group (Table 4). The estimated statistical power of this study was approximately 23%, which underscores its exploratory nature and highlights the need for a larger confirmatory study.

**Table 4:** Corneal Nerve Fiber Abnormalities Observed in Both Groups (Chi-Square; Kruskal–Wallis Test, Holm Corrected *P* Value, and Significance after Holm Correction)

CNF and Matrix Abnormality	CTRL (n)	Ratio (%)	CSCR (n)	Ratio (%)	χ^2^	ddl	*P*	Holm	Significance
Low density	0	0%	8	53%	7.83	1	0.005	0.020	Yes
Neuroma	1	9%	14	93%	16.11	1	<0.001	0.008	Yes
Loops	4	36%	9	69%	3.92	1	0.048	0.144	No
Scalloped	1	9%	12	92%	13.62	1	<0.001	0.008	Yes
Moniliform/bulbs	0	0%	14	100%	20.06	1	<0.001	0.008	Yes
Cell clusters	7	64%	10	77%	3.57	1	0.059	0.144	No
Double edge	5	45%	10	67%	2.40	1	0.121	0.144	No
Dendritic cell	8	73%	15	100%	16.07	1	<0.001	0.008	Yes

CNF = corneal nerve fiber; CSCR = central serous chorioretinopathy; CTRL = control; ddl = degree de liberté/degree of freedom.

## Discussion

Because of its transparency and easy access, the cornea is an ideal tissue for noninvasive analysis of peripheral sensory nerves, which may be altered in ocular and systemic diseases, even in the absence of ocular-associated symptoms.^22^ Although not significant, the mean age of our controls was 7.7 years younger than CSCR. Previous normative studies have consistently demonstrated an age-dependent decline by 65 and beyond in corneal sub-basal nerve parameters.^23^^,^^27^ Although physiological reductions in corneal nerve density have been reported beyond the age of 65, it remains highly unlikely that marked structural abnormalities such as those observed in our study could occur naturally before this age. Furthermore, physiological aging typically impacts nerve fiber density without significantly altering nerve morphology. However, the quantitative reduction of the subepithelial layer must be confirmed with an age-matched CTRL group of patients.

Central serous chorioretinopathy patients had fewer pictures analyzed than CTRL, as exploration was extended, and the total number of pictures analyzed increased when no suspect pattern was detected by the standard round of exploration to ensure no nerve abnormalities were omitted. Conversely, in the CTRL group, the presence of fewer nerve abnormalities led to an increased number of fields explored and imaged, confirming that nerve abnormalities were more frequent and obvious in CSCR patients than in CTRL ones.

The analysis of corneal nerves across all layers in patients with severe chronic CSCR and pachychoroid features revealed morphological indications of corneal nerve abnormalities. These nerve abnormalities were identified by 2 independent graders in different corneal layers, and they were observed more frequently (14/15; 93%) in eyes with CSCR compared with unaffected controls (3/11; 27%), with lesions also being more pronounced in the CSCR group.

In the basal/sub-basal layer, rarefaction was observed in approximately 50% of CSCR patients' corneas, with no difference in nerve tortuosity, which is commonly observed in this age group of patients.^23^ Microneuromas, which may correspond to disorganized and aggregated Schwann cells and fibroblasts surrounding degenerated axon endings,^28^ were frequent in this layer of CSCR corneas and observed in only 1 control cornea. Such microneuromas are common but nonspecific indicators of abnormal axon regeneration. Other signs of nerve pathology were observed, including coiling, looping, twisting of tortuous stromal nerves, stubs of degenerated stromal nerves, and signs of remodeling.^22^ Dendritic cell activation was present in all CSCR corneas but was also frequently observed in control corneas (73%), indicating a lack of specificity and no clear link with pathology.

The frequent and dense nerve abnormalities observed in the corneas of chronic CSCR patients were not associated with any clinically detectable corneal pathology or symptoms in these patients. It is implausible that the observed nerve abnormalities could be a consequence of dry eye disease because the examined patients did not exhibit symptoms of dry eye disease, had no corneal fluorescein staining, and demonstrated normal corneal sensitivity.

The mechanism of corneal nerve abnormalities in chronic CSCR may be related to a broader alteration of peripheral nerves because CSCR patients exhibit functional signs of dysautonomia, measured by heart rate variability and pupillometric responses.^10^^,^^11^ Based on the structural analysis of the choroidal neural network in human eyes, we recently proposed that typical and stable choroidal hyperreflective structures observed around dilated vessels in pachychoroid patients could also correspond to abnormal choroidal nerves,^6^ similar to those observed in rats overexpressing mineralocorticoid receptors and exhibiting a pachychoroid-like phenotype.^6^^,^^29^ As corneal nerves emerge from the choroidal ciliary nerves, the corneal abnormalities could reflect choroidal neuropathy in CSCR. Similarly, corneal nerve abnormalities have been repeatedly reported in patients with autonomic diabetic neuropathy,^30^ where CNF metrics are predictors of incident cardiovascular and cerebrovascular events.^31^ In diabetic patients, corneal confocal imaging is a rapid, noninvasive, and highly sensitive method for diagnosing autonomic diabetic neuropathy.^16^ Evidence of corneal nerve abnormalities has also been described in patients with familial dysautonomia,^16^ leading to neurotrophic keratitis.^32^ Whether corneal nerve abnormalities are early signs and detectable in patients with uncomplicated acute CSCR is currently being evaluated, and their prognostic value should be explored. The presence of nerve abnormalities outside the choroid suggests that choroidal neuropathy is not secondary to CSCR but may contribute to disease pathogenesis. Other factors associated with CSCR, such as sleep apnea,^33^ hypertension, arrhythmia,^34^ or post-traumatic stress disorders,^35^ have also been linked to autonomic nerve dysfunction, which could constitute a unifying link between CSCR and other contributing factors, including systemic or extraocular corticosteroid therapy through corticoid receptor signaling imbalance^25^ and MR-induced choroidal neuropathy.^6^

The mechanisms of autonomic nervous system dysfunction and corneal nerve abnormalities in chronic CSCR remain to be elucidated and may involve genetic and potential environmental/exogenous factors.^36^ Regarding genetic factors, low C4B copy number variation has been strongly associated with severe forms of CSCR,^37^ and C4 plays a major role in monocytic-induced synapse pruning, a mechanism required for neural remodeling. Systemic lipocalin 2 and pentraxin 3, both induced by glucocorticoids,^38^ are significantly reduced in CSCR patients and play important roles in neural remodeling. Genetic and exogenous factors may thus interact and contribute to progressive peripheral neuropathy in chronic CSCR patients, affecting the autonomic nervous system.

Regarding mechanisms of corneal nerve abnormalities, we cannot exclude that they could be secondary to systemic exposure to glucocorticoids. In that case, systemic glucocorticoid exposure could have altered the metabolism of endogenous ocular corticoid^25^ and favored a mineralocorticoid pathway-induced neuropathy, as previously shown in a rat model.^6^ Future studies should control for steroid exposure and comorbidities.

The present study is subject to several limitations. Firstly, its qualitative nature precludes the possibility of deriving quantitative metrics from the data. Secondly, the absence of a validated automatic method to quantify all corneal abnormalities observed with confocal microscopy represents a significant gap in the study's capacity to provide comprehensive analysis. This limitation is mitigated by the blind scoring performed by 2 observers and the comprehensive imaging of the entire corneal surface, which helps reduce bias. Although fewer IVCM images were analyzed in CSCR patients (mean: 658) compared with controls (mean: 1171) because of extended exploration when abnormalities were absent, potentially biasing detection rates, this approach actually enhances confidence that the marked abnormalities observed in CSCR were not inadvertently missed in controls. Indeed, identifying a clear abnormality does not necessitate prolonged exploration, whereas its absence demands increased scrutiny to avoid overlooking subtle anomalies.

A notable shortcoming of the study is the lack of a comprehensive evaluation of the functional parameters of the ocular surface, including lacrimal function, in both the patient cohort and the CTRL group. Addressing this gap in knowledge will require a larger cohort study to determine whether CSCR is associated with any subclinical ocular surface disease. Furthermore, the study's participants were exclusively patients with severe choroidopathy and epitheliopathy. Therefore, our findings apply to severe chronic CSCR cases with pachychoroid features. Because of the limited sample size associated with the rarity of this condition, this study should be regarded as exploratory and hypothesis-generating. Further prospective studies could analyze corneal nerves in patients with acute CSCR and pachychoroid.

## Conclusions

Patients with chronic CSCR exhibit signs of corneal nerve abnormalities, which supports the hypothesis that choroidal neuropathy could contribute to disease pathogenesis. Corneal nerve changes may serve as a noninvasive marker warranting investigation into shared neuropathic mechanisms. Further studies on larger cohorts are needed to confirm these findings.
